# The gut microbiota of wild wintering great bustard (*Otis tarda dybowskii*): survey data from two consecutive years

**DOI:** 10.7717/peerj.12562

**Published:** 2021-11-30

**Authors:** Zhiyuan Lu, Sisi Li, Hongxia Li, Zhucheng Wang, Derong Meng, Jingze Liu

**Affiliations:** 1 Hebei Key Laboratory of Animal Physiology, Biochemistry and Molecular Biology, College of Life Sciences, Hebei Normal University, Shijiazhuang, China; 2 College of Life Sciences, Cangzhou Normal University, Cangzhou, China; 3 Hebei Key Laboratory of Wetland Ecology and Conservation, Hengshui, China

**Keywords:** Great Bustard, Gut microbiota, High-throughput sequencing

## Abstract

**Background:**

The composition of the intestinal microbiota plays a significant role in modulating host health. It serves as a sensitive evaluation indicator and has substantial implications in protecting endangered species. Great Bustards are typical farmland-dependent wintering birds that are highly susceptible to the interference of human activities. However, information regarding their gut microbiota remains scarce.

**Methods:**

To ensure a comprehensive analysis of this crucial data, we collected fecal samples from wild Great Bustards at their wintering habitat for two consecutive years. High-throughput sequencing of the 16S rRNA gene was subsequently applied to characterize their core gut microbiota and determine whether the gut microbial composition was similar or varied interannually.

**Results:**

The gut microbiota of the Great Bustard was primarily comprised of four phyla: Firmicutes (82.87%), Bacteroidetes (7.98%), Proteobacteria (4.49%), and Actinobacteria (3.67%), accounting for 99.01% of the microbial community in all samples. Further analysis revealed 22 genera of core microbes and several pathogens. Notably, there were no significant differences in the alpha-diversity and beta-diversity between the two sample groups from different years.

**Conclusions:**

This study provides essential information for assessing the health and developing targeted protective measures of this threatened species.

## Introduction

The Great Bustard (*Otis tarda dybowskii*) is the largest endangered flying bird globally (Martín et al., 2007). It has been included in the list of vulnerable species in the International Union for Conservation of Nature, globally vulnerable species, key protected birds and umbrella species in grassland habitats, and China’s national Class I key protected animals. It breeds in high-latitude regions, such as Russia, eastern Mongolia, and northeastern China. In winter, the birds migrate south to the middle and lower regions of the Yellow River in China (Kessler et al., 2013). They live in groups and primarily prefer farmlands with flat terrain and short plants as their habitat (Sun et al., 2006; Mi, Huettmann & Guo, 2014; Mi et al., 2017). Food resources are less diverse during winter, with food selection among individuals being more consistent (Liu et al., 2018). These habitats usually have a high traffic of human agricultural activities, including the use of poisonous baits, pesticides, and land-use changes (Lemus et al., 2011; Alonso, 2015), increasing the survival pressure of wintering Great Bustard.

The recent advancements in high-throughput sequencing technologies have enabled reliable analysis of the gut microbial composition (Wu et al., 2009; Faure & Joly, 2015). They have elicited immense research interest on gut microbiota interactions, host physiology, and immune functions in microbial and ecology studies (Grond et al., 2018). The gut microbiota plays a crucial role in regulating the host’s nutrient absorption, detoxification, and immune barriers (Józefiak, Rutkowski & Martin, 2004; Godoy-Vitorino et al., 2008; Kamada et al., 2013). Previous studies on the gut microbiota in wild animals postulate that diet is a key factor that impacts microbiota composition in animals (Muegge et al., 2011) and microbiota composition is heavily dependent on the habitat (Amato et al., 2013). The gut microbiota can be used as a reliable indicator to protect the primary food resources and habitats of endangered species (Bahrndorff et al., 2016). Therefore, understanding the core gut microbiota of wild animals may provide a new approach to conserving the endangered species.

The ability of birds to fly makes them have strong migration and diffusion capacities, high metabolic rate, and relatively short gastrointestinal tracts compared to mammals, which potentially affects the gut microbiota (Guan et al., 2020). Currently, data on the gut microbiota of wild birds are scarce (Grond et al., 2014). Migratory birds have a unique life cycle and often occupy different habitats during different seasons (Somveille, 2016). Their microbial composition could thus vary significantly following seasonal changes (Zhang et al., 2020a). During migration, most birds require continuous energy replenishment in suitable habitats, causing them to interact with complex external environments and consume diverse diets (van Wijk, Bauer & Schaub, 2016; Lewis, Moore & Wang, 2017). Moreover, populations from different breeding and wintering grounds can mingle during temporary rests, thus promoting microbiota exchange between individual animals (Grond et al., 2014; Ryu et al., 2014). The migratory populations can stably inhabit the wintering or breeding ground once the migratory process is complete, raising the question of whether long-term exposure to the same habitats and selection of similar food resources causes the gut microbiota to have a similar composition.

Currently, knowledge of the gut microbiota of the Great Bustard remains scarce. To obtain a more comprehensive evaluation of this crucial data, we collected fecal samples from wild Great Bustards at their wintering habitat for two consecutive years to characterize their core gut microbiota during the wintering period. The study also aimed to determine whether the gut microbial composition of the Great Bustard at specific wintering habitats was similar or varied interannually. This study provides baseline information for developing targeted protective measures of the Great Bustard.

## Materials & Methods

### Sample collection

**Figure 1:**
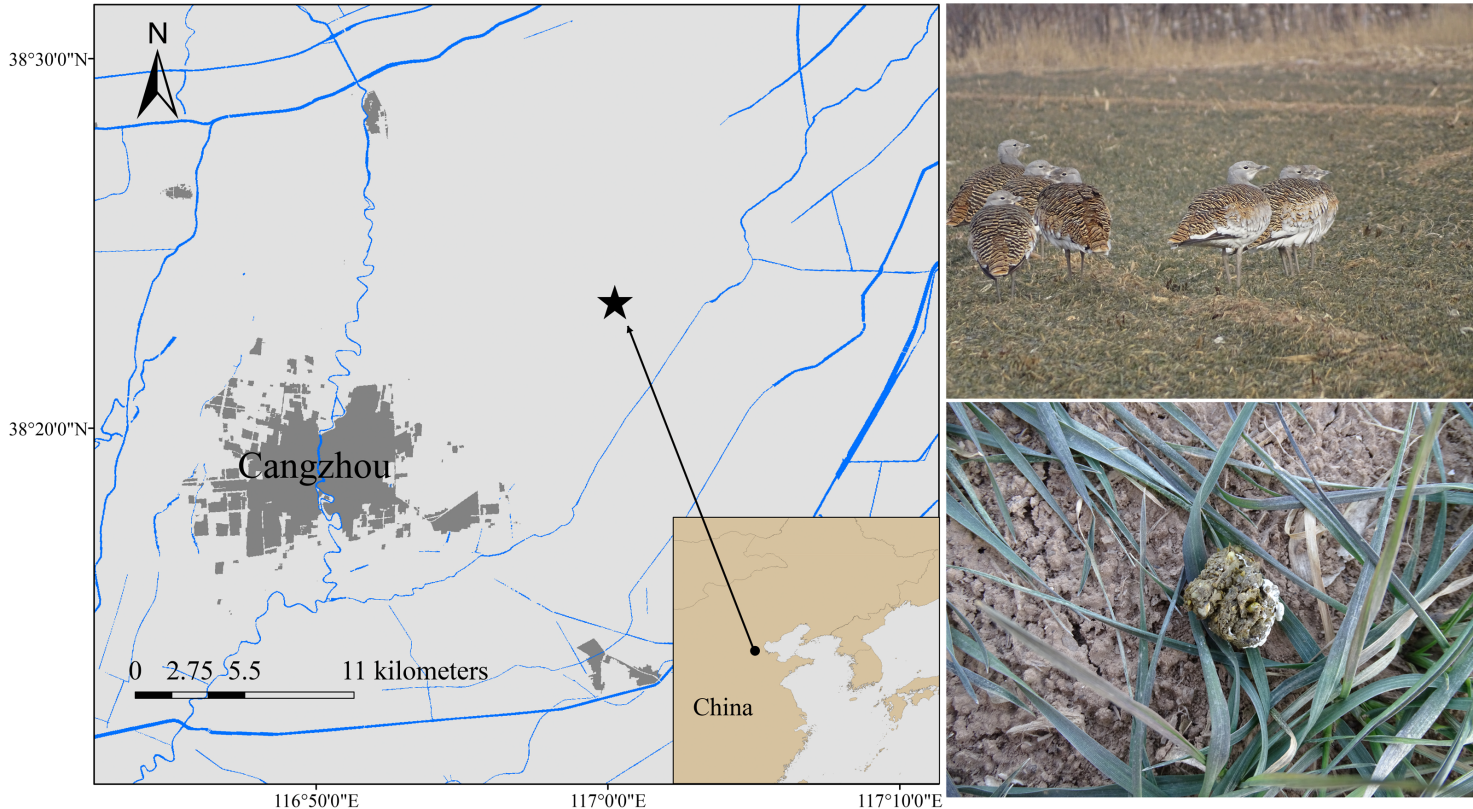
Fecal sampling sites of wild wintering Great Bustard.

Samples were collected from Cangzhou, China (N37°29′–38°57′E 115°42′–117°50′) (Fig. 1). The location is relatively flat, with winter wheat and corn as the main crops, thus making it a vital wintering habitat for the Great Bustard (Mi et al., 2017). We collected 13 fresh fecal samples between December 2019 and February 2020 and labeled them group 1 (EOT 1-EOT 13). Another 22 fresh fecal samples were collected between December 2020 and February 2021 and were labeled group 2 (OT 1-OT 22). The wild Great Bustards were tracked using a high-power monocular telescope. Upon departure of the wild Great Bustards after foraging, the collection was finished using sterile disposable forceps to get fresh fecal samples. The minimum distance between two samples was maintained at 5 m to avoid collecting multiple samples from the same individual. The middle portions of the feces were sampled into 15 mL sterile centrifuge tubes, transported in a −20 °C portable freezer, and stored at −80 °C before processing.

### DNA extraction, amplification, and sequencing

Total DNA in the fecal sample was extracted using the OMEGA-soil DNA kit (Omega Bio-Tek, USA), followed by a quantity and quality check using a NanoDrop2000 (NanoDrop Technologies, USA). The DNA quality was further verified on a 1% agarose gel by electrophoresis. PCR amplification of the V3–V4 variable region of the 16S rRNA was then conducted on an ABI GeneAmp^®^ 9700, USA, thermocycler using the 338 F (5′-ACTCCTACGGGAGGCAGCAG-3′) and 806 R (5′-GGACTACHVGGGTWTCTAAT-3′) primers (Mori et al., 2014). The cycling conditions were initial denaturation at 95 °C for 3 min, followed by 27 cycles of denaturation, annealing, and extension at 95 °C for 30 s, 55 °C for 30 s, and 72 °C for 45 s, respectively, and a final extension at 72 °C for 10 min. The PCR reaction mix contained 4 µL of 5 × FastPfu buffer, 2 µL of 2.5 mM dNTPs, 0.8 µL forward primer (5 µM), 0.8 µL reverse primer (5 µM), 0.4 µL FastPfu DNA polymerase, and 10 ng DNA template in a total volume of 20 µL. The PCR products were run on a 2% gel and then subsequently extracted, purified using the AxyPrep DNA Gel Extraction Kit (Axygen Biosciences, Union City, CA, USA), eluted with Tris–HCl, and quantified using a 2% agarose gel and QuantiFluor^TM^-ST (Promega, Madison, WI, USA). Library preparation and high-throughput sequencing were performed on an Illumina MiseqPE 300 platform according to the standard protocols by Majorbio Bio-Pharm Technology Co., Ltd. (Shanghai, China).

### Data processing and analysis

Raw sequences were subjected to quality control analysis using the Fastp v0.19.6 software and merged using FLASH v1.2.11. The 300 bp reads were truncated at any site receiving an average quality score < 20 over a 50 bp sliding window. The truncated reads shorter than 50 bp, and those containing ambiguous characters were discarded. Only those overlapping sequences exceeding 10 bp were assembled. The maximum mismatch rate for overlapping areas was set at 0.2. Reads that could not be assembled were discarded. The samples were distinguished using barcodes and primers and adjusted for sequence orientation with exact barcode matches and a maximum of two nucleotide mismatches in primer matches. Sequences that met the quality control threshold were clustered into operational taxonomic units (OTUs) at a 97% similarity threshold using the UPARSE v7.1 software. Species classification and annotation of each sequence were done using the RDP classifier v11.5 and compared using the Silva database (SSU138) at a 70% threshold. Rarefaction curves were subsequently plotted based on each sample’s observed richness (Sobs) to evaluate the sequencing efficiency. Alpha-diversity indexes, including community richness (Chao1), community evenness (Shannoneven), community diversity (Shannon), and community coverage (Good’s Coverage), were calculated using Mothur v1.30.2 based on the OTUs. The alpha-diversity indexes between groups were compared using the non-parametric Mann–Whitney *U* test at a significance threshold of *P* < 0.05. Beta-diversity was described based on the bray-curtis distance using the principal co-ordinates analysis (PCoA). Sample dissimilarities were subsequently analyzed by Analysis of Similarities (ANOSIM) using 999 random permutations at a significance threshold of *P* < 0.05. Core microbes were those with >1% abundance and present in >50% of the samples (Grond et al., 2018). The functions of the OTUs in each sample were analyzed using PICRUSt 2 set at default following the Kyoto Encyclopedia of Genes and Genomes orthologs and Enzyme Commission numbers (Douglas et al., 2020).

## Results

### Sequencing and alpha-diversity analysis

**Figure 2:**
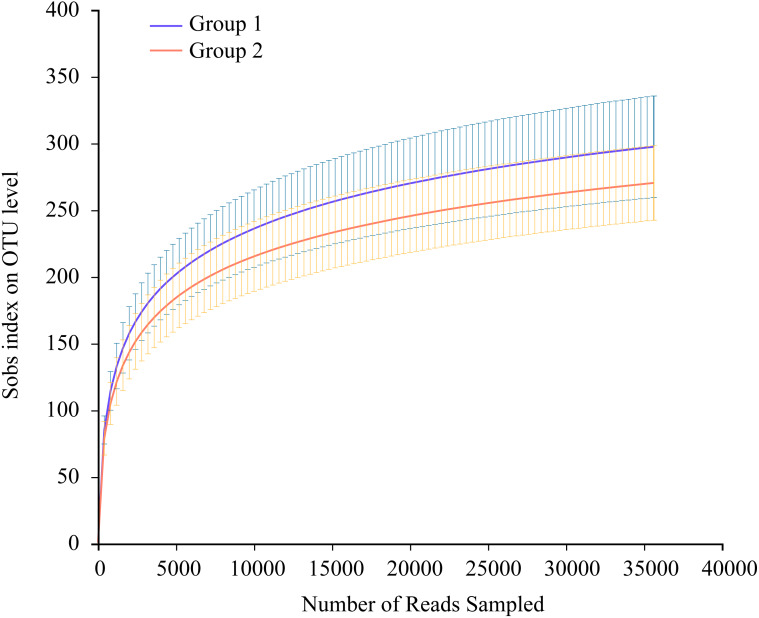
Average rarefaction curve representing variation in the Sobs index at increasing sequencing depth (35,644 reads only) of two groups. The error bars on the Sobs index correspond to the lower and upper bound 97% confidence intervals.

Thirty-five Great Bustard fecal samples were collected in two consecutive wintering periods. The samples yielded 1,923,278 sequences after quality control. The mean number of all sequences in the samples was 54,951; 40,785 for EOT 13 and 74,301 for OT 7, with a length of 405–415 bp (Table S1). All data were processed based on the minimum number of sample sequences to avoid statistical differences caused by different sequencing depths. There were 767 OTUs belonging to 18 phyla, 34 classes, 90 orders, 144 families, and 279 genera. The rarefaction curves suggested that the sequencing amounts were adequate to reflect most microbial diversity information in all samples (Fig. 2).

Table S2 outlines the alpha-diversity analysis of the gut microbiota in wild wintering Great Bustard. The Chao 1 index revealed the varying community richness in the 35 samples comprising 266–436 OTUs. The OT 20 sample had the lowest richness, whereas the EOT 3 sample had the highest richness. Notably, the Shannon index denoted a measure of community diversity, with the OT 1 sample exhibiting the lowest diversity and the EOT 3 sample the highest diversity. The Shannoneven index represented the community evenness, with the OT 1 and OT 17 samples exhibiting the lowest and highest evenness, respectively. The Good’s Coverage highlighting the coverage of each sample library revealed > 0.99 coverage in all samples, indicating that the sequencing results were a true reflection of the microbiota population in the samples.

### Microbial composition and relative abundance

**Figure 3:**
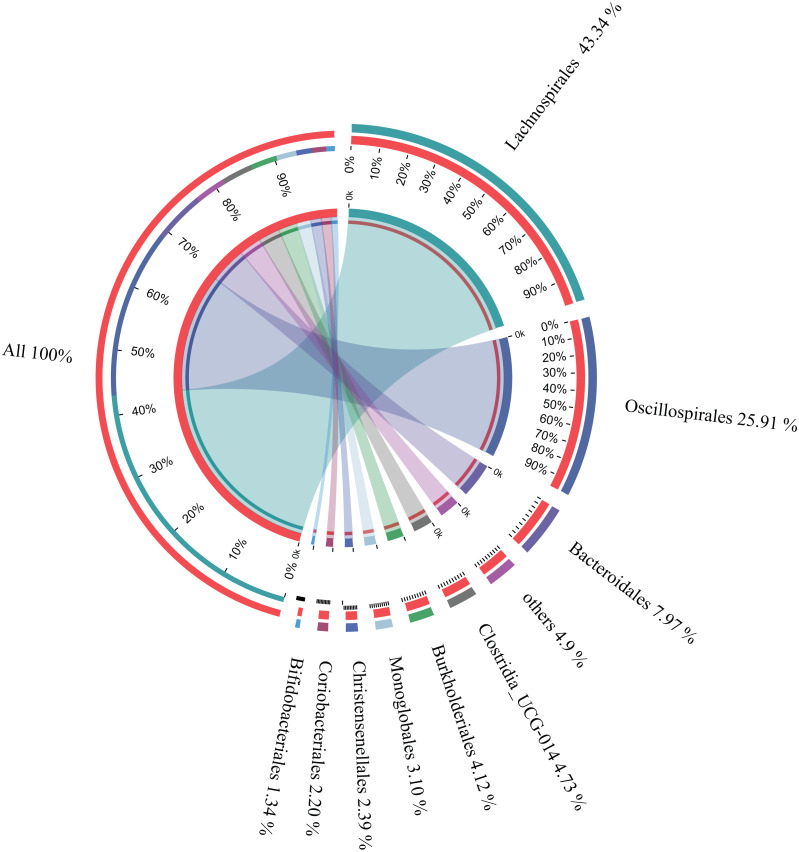
Relative abundances of core bacterial orders of wild wintering Great Bustard.

The microbial composition of the 35 samples was analyzed, and their mean relative abundance at the phylum level was subsequently calculated. The core phyla were: Firmicutes (82.87%), Bacteroidetes (7.98%), Proteobacteria (4.49%), and Actinobacteria (3.67%), accounting for 99.01% of the total microbial composition in all samples. Moreover, there were nine core orders, including Lachnospirales (43.34%), Oscillospirales (25.91%), Bacteroidales (7.97%), Clostridia UCG-014 (4.73%), Burkholderiales (4.12%), Monoglobales (3.10%), Christensenellales (2.39%), Coriobacteriales (2.20%), and Bifidobacteriales (1.34%), accounting for 95.1% of the total microbial composition in all samples (Fig. 3). In addition, 22 core genera constituting 83.48% of the microbial composition in all samples were identified (Table 1). These findings suggested that these were the core microbes in wintering Great Bustard.

**Table 1:** The relative abundance of core genera in the gut microbial communities of wild wintering Great Bustard.

**Genus**	**%of total**	**Phylum**	**Class**	**Order**	**Family**
*Butyrivibrio*	15.46	Firmicutes	Clostridia	Lachnospirales	Lachnospiraceae
*unclassified_f__Lachnospiraceae*	14.05	Firmicutes	Clostridia	Lachnospirales	Lachnospiraceae
*Oscillibacter*	5.93	Firmicutes	Clostridia	Oscillospirales	Oscillospiraceae
*Ruminococcus_torques_group*	4.85	Firmicutes	Clostridia	Lachnospirales	Lachnospiraceae
*Clostridia UCG-014*	4.73	Firmicutes	Clostridia	Clostridia UCG-014	Clostridia UCG-014
*unclassified_f__Ruminococcaceae*	4.50	Firmicutes	Clostridia	Oscillospirales	Ruminococcaceae
*Subdoligranulum*	4.32	Firmicutes	Clostridia	Oscillospirales	Ruminococcaceae
*Alistipes*	3.91	Bacteroidetes	Bacteroidia	Bacteroidales	Rikenellaceae
*Marvinbryantia*	3.59	Firmicutes	Clostridia	Lachnospirales	Lachnospiraceae
*Burkholderia-Caballeronia-Paraburkholderia*	3.29	Proteobacteria	Gammaproteobacteria	Burkholderiales	Burkholderiaceae
*Monoglobus*	3.10	Firmicutes	Clostridia	Monoglobales	Monoglobaceae
*Christensenellaceae_R-7_group*	2.37	Firmicutes	Clostridia	Christensenellales	Christensenellaceae
*UBA1819*	1.84	Firmicutes	Clostridia	Oscillospirales	Ruminococcaceae
*norank_f__Ruminococcaceae*	1.65	Firmicutes	Clostridia	Oscillospirales	Ruminococcaceae
*Bacteroides*	1.41	Bacteroidetes	Bacteroidia	Bacteroidales	Bacteroidaceae
*Bifidobacterium*	1.34	Actinobacteria	Actinobacteria	Bifidobacteriales	Bifidobacteriaceae
*Tyzzerella*	1.33	Firmicutes	Clostridia	Lachnospirales	Lachnospiraceae
*Enterorhabdus*	1.32	Actinobacteria	Coriobacteriia	Coriobacteriales	Eggerthellaceae
*Eubacterium siraeum group*	1.30	Firmicutes	Clostridia	Oscillospirales	Ruminococcaceae
*Anaerostipes*	1.09	Firmicutes	Clostridia	Lachnospirales	Lachnospiraceae
*UCG-005*	1.08	Firmicutes	Clostridia	Oscillospirales	Oscillospiraceae
*Ruminococcus*	1.02	Firmicutes	Clostridia	Oscillospirales	Ruminococcaceae

### Comparison of microbial abundance between different periods

**Figure 4:**
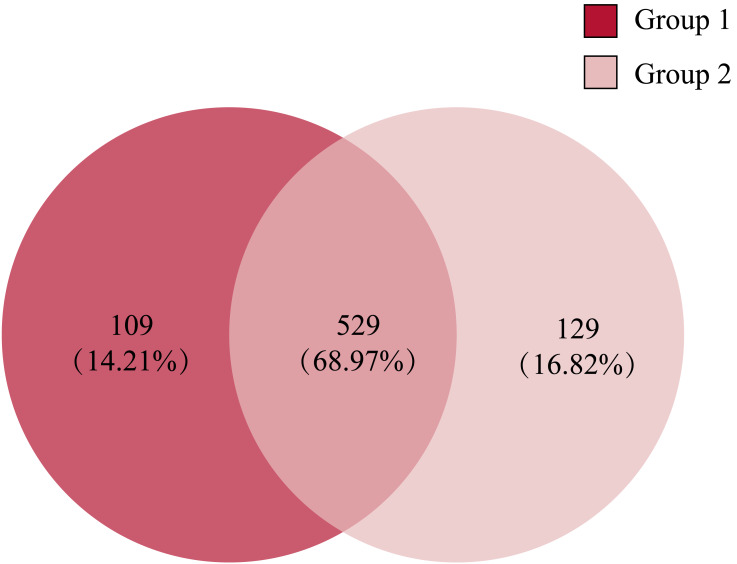
The Venn diagram of the OTUs in all fecal samples among groups.

**Figure 5:**
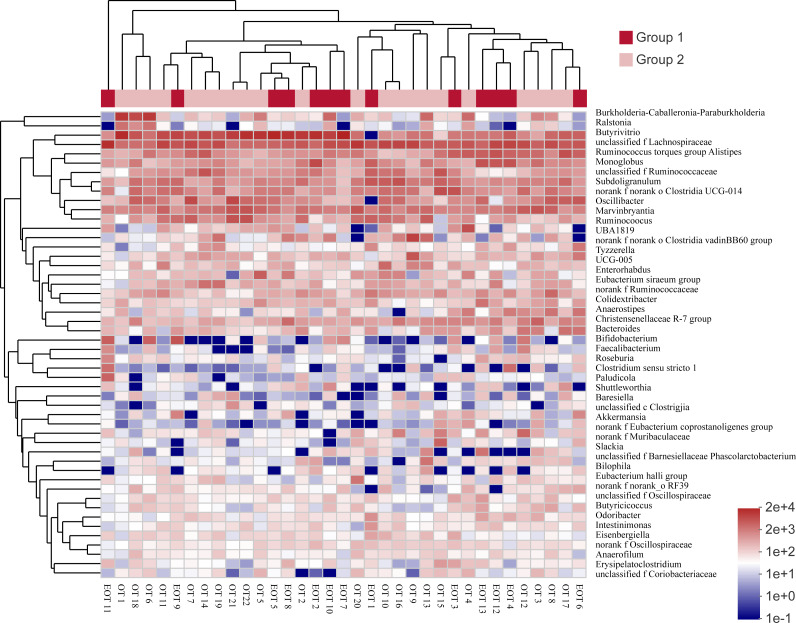
The heat map of species abundance in different genera. Samples information is transversely listed, and species annotations are longitudinally shown. The left clustering tree is a species-related clustering tree, and the upper tree is a sample-related clustering tree based on the average clustering algorithm. The heat map was performed by discrepancies of species abundance between samples, with colors gradually changed from deep red to deep blue, by high species abundance to low.

Comparing the gut microbiota of Great Bustard fecal samples collected at different wintering periods revealed common and unique microbial populations in both groups (Fig. 4). There were 529 common OTUs between the two groups and 109 and 129 unique OTUs in groups 1 and 2, respectively. Figure 5 shows the heatmap analysis of the top 50 most abundant genera in the two sample groups and their clustering based on genus and inter-sample abundance. Sample clustering was not affected by interannual variation.

**Figure 6:**
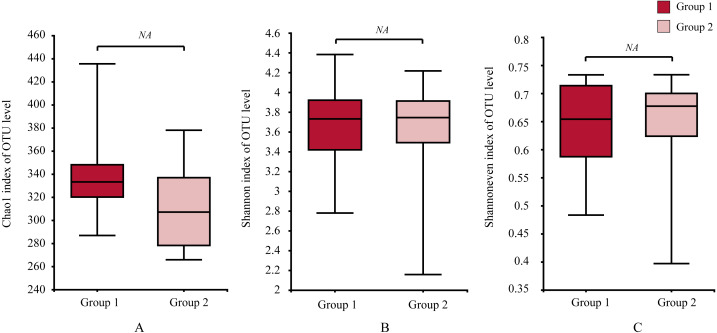
Differences of alpha diversity between two groups. (A) the Chao1 estimator, (B) Shannon diversity indexes, and (C) Shannon index-based measure of evenness. Wilcoxon rank-sum test for an estimator for different groups significant, NA means *p* > 0.05.

**Figure 7:**
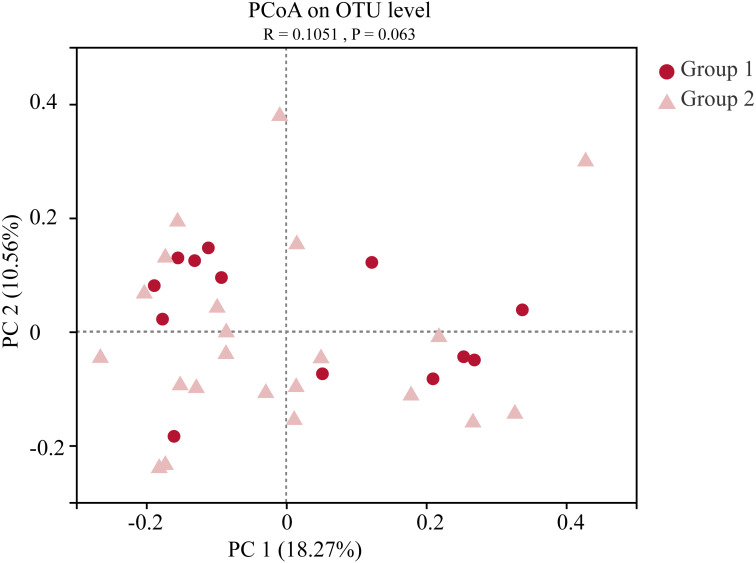
Principal co-ordinates analysis (PCoA) of the gut microbial communities of wild wintering Great Bustard. Differences between the two groups were analyzed by Analysis of similarities (ANOSIM) with 999 permutations.

**Figure 8:**
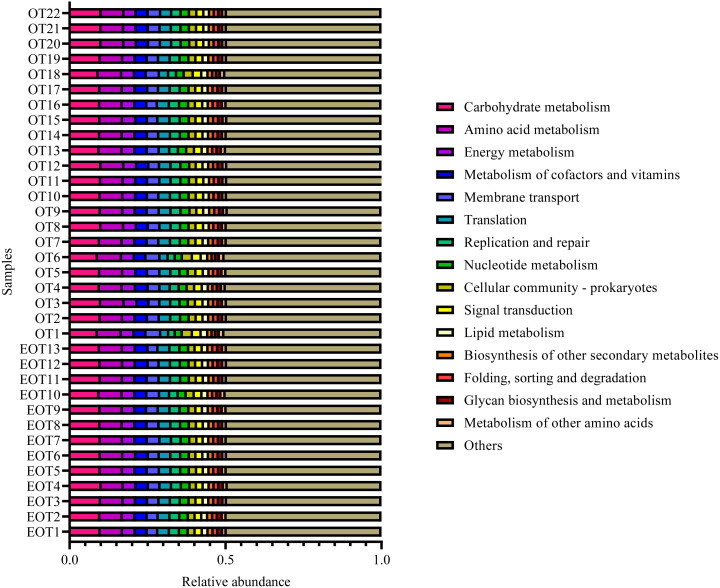
Predicted microbial functions using PICRUSt 2.

Moreover, the alpha-diversity analysis revealed insignificant differences in community richness (Chao 1), evenness (Shannoneven), and diversity (Shannon) (Fig. 6) between groups 1 and 2 (Table S3). PCoA analysis revealed that the two sample groups had a high microbial composition similarity (Fig. 7). The level 2 functional prediction of 15 microbial populations with the highest relative abundance in the two groups revealed similar functions between the two groups. The PICRUSt 2 mediated prediction suggested their involvement in carbohydrate metabolism, amino acid metabolism, energy metabolism, metabolism of cofactors and vitamins, membrane transport, translation, replication and repair, and nucleotide metabolism (Fig. 8).

## Discussion

In this study, the gut microbiota of the Great Bustard in the same wintering habitat was investigated for two consecutive years by analyzing their 16S rRNA gene sequences. The Great Bustard is a long-distance migratory bird that stays in its wintering habitat for about four months each year (Kessler et al., 2013). The gut microbiota may be affected by other uncertain factors besides birds living in the same habitat a year long. The core gut microbiota can thus be more comprehensively described using survey data from different years. In addition, the uncontrollable variations between individuals are a common challenge for gut microbiome studies in wild birds (Wang et al., 2019; Wang et al., 2020). Compared with previous studies of this threatened species (Liu et al., 2020), this study employed a larger sample size to improve the accuracy of the partial results.

Great bustards have an extensive food selection, with varying food compositions in different seasons. They mostly consume animal-based diets in the summer and crops during the winter season (Liu et al., 2018; Faragó, 2019; Gong et al., 2019). Migratory birds usually have significantly different gut microbiota between seasons (Zhang et al., 2020a). There were no significant differences in diversity, evenness, and richness indices between the two sample groups. PCoA analysis further indicated that both groups had a highly similar microbial composition. Long-term exposure to the same habitats and selection of similar food resources led to similar gut microbiota composition. Field investigations conducted over many years have revealed that Great bustards live in groups during the wintering period. Group living habits allow individual animals to develop the same temporal rhythm, share a common foraging space, and facilitate inter-individual exchanges. These behaviors are favorable for the consistent changes in the gut microbiota within this species. This result was largely consistent with previous studies, which report that Great Bustards from wintering habitats with differing diet modes (wheat-corn and rice-peanut) have different gut microbial compositions. These findings collectively suggest that diet is potentially a significant microbial composition determinant in Great Bustards (Liu et al., 2020).

During winter, the gut microbiota of Great Bustard comprised four main phyla: Firmicutes (82.87%), Bacteroidetes (7.98%), Proteobacteria (4.49%), and Actinobacteria (3.67%), accounting for 99.01% of the total microbial composition. Firmicutes was the predominant phylum in all samples, with a high Firmicutes/Bacteroidetes ratio. A correlation between human weight and gut microbiota revealed that obese subjects have increased Firmicutes and decreased Bacteroidetes. A reduction in the Firmicutes/Bacteroidetes ratio is thus directly associated with weight loss (Ley et al., 2006). Due to the low temperature and food resource scarcity during winter, maintaining a high Firmicutes/Bacteroidetes ratio may help improve the energy acquisition efficiency of Great Bustards during winter because of the low temperature and food scarcity. In our survey area, the Great Bustards primarily consumed corn, wheat, and soybeans seeds scattered after harvest and fresh winter wheat seedlings in the farmlands during the wintering period (Liu et al., 2018). Previously studies postulate that supplementing corn starch in the diet increases the overall abundance of Firmicutes and Bacteroidetes in poultry cecum (Zhang et al., 2020b). Bacteroidetes are common in herbivorous birds and are known to enhance the hydrolysis of polysaccharides, cellulose, and other complex polymers (Thomas et al., 2011).

In this study, we identified 22 core genera in the gut microbiota of Great Bustard, which played an essential role in the degradation of plant cellulose and starch. Microbial species belonging to the *Butyrivibrio* genus, which were the core microorganism with the highest abundance (15.46%), have been extensively reported in the rumen and colon of animals. A study on the effect of different grasses on the composition of the ruminal microbiota in dairy cows revealed that *Butyrivibrio* and *Ruminococcaceae* species are highly abundant during grass degradation (Liu et al., 2016). *Butyrivibrio* degrades cellulose into short-chain fatty acids (Paillard et al., 2007; Donohoe et al., 2011). Similarly, genetic analyses have postulated that members of the Lachnospiraceae and Ruminococcaceae families also degrade plant cellulose into short-chain fatty acids (Biddle et al., 2013). The *Christensenellaceae_R-7_group* (2.37%) also possesses a similar function (Long & Venema, 2020). These results further confirm that the gut microbiome is closely related to the composition of the host diet. Notably, this study found a few potential pathogens, including *Escherichia* coli, *Helicobacter* sp., and *Streptococcus* sp., in low abundance in some samples. Though these pathogens are commonly found in wild birds (Fu et al., 2020; Balta et al., 2021), changes in their abundance should be further examined to determine their significance in the health of Great Bustards during the wintering period.

## Conclusion

This study successfully characterized the core gut microbes of Great Bustard by high-throughput sequencing of their 16S rRNA gene. The highly abundant core microbes were closely associated with the foods the birds consumed during winter. Nonetheless, several common pathogens were also identified in the gut microbiota of the Great Bustards. Changes in the abundance of these pathogens can serve as a warning sign towards protecting these endangered species. Future studies should integrate other techniques, such as metagenomics and metabonomics, to understand the function and mechanisms of the gut microbiota.

## Supplemental Information

10.7717/peerj.12562/supp-1 Supplemental Information 1 The sequencing information of each sample

10.7717/peerj.12562/supp-2 Supplemental Information 2 Alpha diversity index of each sample

10.7717/peerj.12562/supp-3 Supplemental Information 3 Non-parametric Mann–Whitney *U* test was used to test the Alpha-diversity index significance

## Additional Information and Declarations

### Data Availability

The following information was supplied regarding data availability: The raw data is available at NCBI: PRJNA719683, PRJNA720495. The data are also available at figshare: lu, zhiyuan (2021): Sequence Data.zip. figshare. Dataset. https://doi.org/10.6084/m9.figshare.14897052.v1
