# Supplementary material for: The gut microbiota of wild wintering great bustard (Otis tarda dybowskii): survey data from two consecutive years
Source: PeerJ. 2021 Nov 30;9:e12562. doi: 10.7717/peerj.12562 (PMC8641483; doi:10.7717/peerj.12562)
Supplement: Supplemental Information 1 [file peerj-09-12562-s001.docx]

| Sample | Seq_num | Base_num | Mean_length | Min_length | Max_length |
| --- | --- | --- | --- | --- | --- |
| OT 1 | 42624 | 17640194 | 413.8559 | 401 | 430 |
| OT 9 | 70669 | 28651246 | 405.4288 | 211 | 430 |
| OT 10 | 67156 | 27279903 | 406.2169 | 231 | 490 |
| OT 11 | 64920 | 26333118 | 405.6241 | 244 | 431 |
| OT 12 | 70569 | 28873712 | 409.1557 | 282 | 431 |
| OT 13 | 45613 | 18775870 | 411.6342 | 265 | 510 |
| OT 14 | 63298 | 25724731 | 406.4067 | 230 | 431 |
| OT 15 | 66975 | 27306358 | 407.7097 | 235 | 490 |
| OT 16 | 68007 | 27660696 | 406.7331 | 229 | 494 |
| OT 17 | 71229 | 29086954 | 408.3583 | 228 | 491 |
| OT 18 | 45472 | 18725902 | 411.8117 | 399 | 471 |
| OT 2 | 55451 | 22558119 | 406.8118 | 216 | 540 |
| OT 19 | 51272 | 20844628 | 406.5499 | 245 | 500 |
| OT 20 | 55707 | 22628977 | 406.2142 | 258 | 512 |
| OT 21 | 56604 | 22982325 | 406.0195 | 219 | 430 |
| OT 22 | 55055 | 22318009 | 405.3766 | 210 | 430 |
| OT 3 | 51483 | 21091039 | 409.67 | 345 | 430 |
| OT 4 | 41093 | 16756108 | 407.7606 | 378 | 430 |
| OT 5 | 45569 | 18527145 | 406.5734 | 378 | 513 |
| OT 6 | 41961 | 17414459 | 415.0153 | 401 | 431 |
| OT 7 | 74301 | 30171139 | 406.0664 | 283 | 430 |
| OT 8 | 58245 | 23683133 | 406.6123 | 343 | 511 |
| EOT 12 | 43874 | 17980010 | 409.8101 | 214 | 430 |
| EOT 13 | 40785 | 16761428 | 410.9704 | 248 | 434 |
| EOT 11 | 49779 | 20254151 | 406.8814 | 336 | 488 |
| EOT 1 | 53112 | 21707385 | 408.7096 | 239 | 430 |
| EOT 10 | 41065 | 16801609 | 409.1467 | 262 | 430 |
| EOT 2 | 49887 | 20405319 | 409.0308 | 254 | 497 |
| EOT 3 | 55913 | 22773493 | 407.3023 | 221 | 500 |
| EOT 4 | 57545 | 23528123 | 408.8648 | 276 | 431 |
| EOT 5 | 44502 | 18039801 | 405.3706 | 270 | 511 |
| EOT 6 | 62311 | 25433715 | 408.1738 | 231 | 479 |
| EOT 7 | 60368 | 24542914 | 406.555 | 245 | 490 |
| EOT 8 | 55849 | 22687419 | 406.2278 | 305 | 489 |
| EOT 9 | 45015 | 18377260 | 408.2475 | 232 | 490 |

Table S1 The sequencing information of each sample
