# Supplementary material for: The gut microbiota of wild wintering great bustard (Otis tarda dybowskii): survey data from two consecutive years
Source: PeerJ. 2021 Nov 30;9:e12562. doi: 10.7717/peerj.12562 (PMC8641483; doi:10.7717/peerj.12562)
Supplement: Supplemental Information 2 [file peerj-09-12562-s002.docx]

Table S2 Alpha diversity index of each sample

| Samples | Chao 1 | shannoneven | shannon | coverage |
| --- | --- | --- | --- | --- |
| OT 1 | 274.2308 | 0.397012 | 2.157251 | 0.998625 |
| OT 9 | 316.4444 | 0.623059 | 3.530517 | 0.998906 |
| OT 10 | 311.625 | 0.701386 | 3.926656 | 0.998962 |
| OT 11 | 338.1304 | 0.676205 | 3.82697 | 0.998625 |
| OT 12 | 353.75 | 0.708596 | 4.046383 | 0.998709 |
| OT 13 | 298.3871 | 0.69584 | 3.882598 | 0.998709 |
| OT 14 | 370.6667 | 0.652118 | 3.736701 | 0.998653 |
| OT 15 | 269.1613 | 0.682204 | 3.750189 | 0.998878 |
| OT 16 | 291.9545 | 0.705182 | 3.921295 | 0.998934 |
| OT 17 | 365.25 | 0.733213 | 4.215532 | 0.99885 |
| OT 18 | 268 | 0.679788 | 3.73691 | 0.999074 |
| OT 2 | 271.5385 | 0.641379 | 3.566507 | 0.999299 |
| OT 19 | 318.8966 | 0.678425 | 3.851253 | 0.998878 |
| OT 20 | 265.8077 | 0.655561 | 3.553494 | 0.998709 |
| OT 21 | 367.6 | 0.560759 | 3.096211 | 0.998625 |
| OT 22 | 315.5 | 0.503899 | 2.778211 | 0.998457 |
| OT 3 | 378 | 0.722372 | 4.173613 | 0.998457 |
| OT 4 | 302.5313 | 0.681153 | 3.850211 | 0.999046 |
| OT 5 | 268.875 | 0.587564 | 3.215309 | 0.998906 |
| OT 6 | 302.1429 | 0.615801 | 3.436005 | 0.998878 |
| OT 7 | 290.2174 | 0.626196 | 3.477243 | 0.998906 |
| OT 8 | 333.037 | 0.721791 | 4.099899 | 0.998681 |
| EOT 12 | 292.2308 | 0.71384 | 3.915265 | 0.998962 |
| EOT 13 | 320.0588 | 0.721598 | 4.073742 | 0.99899 |
| EOT 11 | 299 | 0.636873 | 3.541448 | 0.998906 |
| EOT 1 | 286.7941 | 0.682806 | 3.809872 | 0.998906 |
| EOT 10 | 348.1304 | 0.654077 | 3.724139 | 0.998625 |
| EOT 2 | 333.4 | 0.574537 | 3.261503 | 0.998709 |
| EOT 3 | 435.5263 | 0.732954 | 4.382248 | 0.998429 |
| EOT 4 | 331.6071 | 0.690121 | 3.920009 | 0.998681 |
| EOT 5 | 328.3871 | 0.54722 | 3.112026 | 0.998709 |
| EOT 6 | 333.1579 | 0.723668 | 4.10311 | 0.99885 |
| EOT 7 | 392.12 | 0.483312 | 2.778751 | 0.998233 |
| EOT 8 | 380.9767 | 0.58731 | 3.418194 | 0.998261 |
| EOT 9 | 345.0476 | 0.651797 | 3.730617 | 0.99885 |
