# Supplementary material for: The gut microbiota of wild wintering great bustard (Otis tarda dybowskii): survey data from two consecutive years
Source: PeerJ. 2021 Nov 30;9:e12562. doi: 10.7717/peerj.12562 (PMC8641483; doi:10.7717/peerj.12562)
Supplement: Supplemental Information 3 [file peerj-09-12562-s003.docx]

Table S3 Non-parametric Mann-Whitney U test was used to test the Alpha-diversity index significance

| Estimators | Group 1-Mean | Group 1-Sd | Group 2-Mean | Group 2-Sd | Pvalue | Qvalue |
| --- | --- | --- | --- | --- | --- | --- |
| shannon | 3.6747 | 0.44337 | 3.6286 | 0.4818 | 1 | 1 |
| chao1 | 340.5 | 41.929 | 312.35 | 36.999 | 0.05375 | 0.09406 |
| shannoneven | 0.64616 | 0.077551 | 0.6477 | 0.07946 | 1 | 1 |
